# ADD3 Deletion in Glioblastoma Predicts Disease Status and Survival

**DOI:** 10.3389/fonc.2021.717793

**Published:** 2021-12-14

**Authors:** Karrie Mei-Yee Kiang, Stella Sun, Gilberto Ka-Kit Leung

**Affiliations:** Department of Surgery, Li Ka Shing Faculty of Medicine, The University of Hong Kong, Queen Mary Hospital, Hong Kong, Hong Kong SAR, China

**Keywords:** loss of heterozygosity, glioblastoma, deletion mapping, tumor suppressor, ADD3

## Abstract

Loss of heterozygosity (LOH) on chromosome 10 frequently occurs in gliomas. Whereas genetic loci with allelic deletion often implicate tumor suppressor genes, a putative tumor suppressor Adducin3 (*ADD3*) mapped to chromosome 10q25.2 was found to be preferentially downregulated in high-grade gliomas compared with low-grade lesions. In this study, we unveil how the assessment of *ADD3* deletion provides clinical significance in glioblastoma (GBM). By deletion mapping, we assessed the frequency of LOH in forty-three glioma specimens using five microsatellite markers spanning chromosome 10q23-10qter. Data were validated in The Cancer Genome Atlas (TCGA) cohort with 203 GBM patients. We found that allelic loss in both D10S173 (*ADD3/MXI1* locus) and D10S1137 (*MGMT* locus) were positively associated with tumor grading and proliferative index (*MIB-1*). However, LOH events at only the *ADD3/MXI1* locus provided prognostic significance with a marked reduction in patient survival and appeared to have diagnostic potential in differentiating high-grade gliomas from low-grade ones. Furthermore, we showed progressive loss of *ADD3* in six out of seven patient-paired gliomas with malignant progression, as well as in recurrent GBMs. These findings suggest the significance of *ADD3/MXI1* locus as a promising marker that can be used to refine the LOH10q assessment. Data further suggest the role of *ADD3* as a novel tumor suppressor, whereby the loss of *ADD3* is indicative of a progressive disease that may at least partially account for rapid disease progression in GBM. This study revealed for the first time the downregulation of *ADD3* on the genetic level resulting from copy number deletion.

## Introduction

Glioblastoma (GBM), the most malignant primary brain tumor in adults, is often characterized by high levels of genomic instability. Loss of heterozygosity (LOH) of chromosome 10q is among the most frequent genetic alterations that occur in over 80% of *de novo* cases and 60%–70% of secondary cases, but it is less frequent in anaplastic astrocytoma (40%) and rare in low-grade gliomas (1–4). Other frequent abnormalities in primary GBM include *EGFR* amplification (36%), *p16^INK4a^* deletion (31%), *TP53* mutation (28%), and *PTEN* mutation (25%) (5, 6). The assessment of LOH10q genotype is not included in the recent WHO classification for gliomas (WHO 2016), as it is believed that the assessment of both chromosome 7+/10q− and *TERTp* mutations is perhaps more informative than assessing *EGFR* amplification for the diagnosis of GBM (7). Moreover, most studies to date have identified LOH of 10q as a poor prognostic marker for high-grade gliomas (8–10). Allelic deletion on chromosome 10q has also been observed in various advanced human malignancies (11–14). This suggests that such allelic regions might harbor tumor suppressor genes and be involved in the development of cancer.

Allelic deletions can occur in the entire copy or part of chromosome 10. Several commonly deleted loci have been identified on chromosome 10q in the region spanning 10q23 to 10qter, encompassing the allelic loci of a number of genes with well-established tumor-suppressive roles (e.g., *MMAC/PTEN*, *SUFU*, *FGFR2*, and *DMBT1*) or putative tumor-suppressive roles (e.g., *ADD3/MXI1*, *LGI1*, and *BTRC*) (9, 15–17). When comparing cohorts with primary and secondary gliomas, the acquisition of a malignant phenotype with marked proliferative activity was observed in a deletion mapping on 10q25-qter (18). This further suggests the importance of tumor suppressor genes in 10q25-qter apart from *PTEN* that are likely to be involved in the malignant progression of glioma.

Among these putative tumor suppressor genes, *ADD3* was found to be downregulated in high-grade gliomas when compared with its less malignant counterpart in several gene expression profiling studies (19–21). *ADD3* is located on chromosome 10q25.1-25.2, which is known to be a functional tumor suppressor region. While it primarily functions as a cytoskeleton protein, *ADD3* is characterized as a negative regulator of tumor growth and is negatively associated with malignant phenotypes such as angiogenesis in GBM (22). However, the basis behind *ADD3* downregulation has not been identified for which both genetic and epigenetic modifications should be taken into consideration when studying its functional characteristics in cancer cells. Moreover, although *PTEN* locus is often tested for chromosome LOH10q status, the frequency of allelic deletion on other 10q loci has not been adequately addressed in previous studies.

Here, we performed deletion mapping analysis in a glioma cohort to determine the regions of allelic loss in chromosome 10q and to establish correlations between their accumulation and different pathological phenotypes. By correlating patient characteristics to the occurrence of allelic loss, we determined whether the aberrant expression of *ADD3* in malignant glioma results from LOH10q, which may be involved in tumor relapse and malignant progression. We will also evaluate and highlight the potential application of *ADD3* as a novel biomarker for diagnostic, prognostic, and future therapeutic implications.

## Materials and Methods

### Human Glioma Tissue Specimens

Fresh tumor tissues were snap-frozen in liquid nitrogen immediately after surgical resection at our institution. All specimens were obtained with informed consent from the patients. The study protocol was approved by our Institution Review Board. Diagnosis and histological classification were confirmed by specialists according to the WHO’s brain tumor classification system before the release of the WHO 2016 classification on gliomas. Forty-three glioma specimens with WHO grades 2–4 (8 grade 2, 12 grade 3, and 23 GBM/grade 4) were collected for clinical and molecular analyses in the study. Specimens were obtained at the time of surgery between 2009 and 2016 and were stored at −80°C until prior study use. All diagnoses were made radiologically *via* MRI before surgery and confirmed histologically by a certified pathologist. Tumors from another cohort of ten patients (P1–P10) who developed recurrence after the initial surgical treatment of the disease were also collected during the second operation for longitudinal comparison in our study.

### Clinical Data

The records of forty-three patients were retrieved from their medical records from initial consultation to the latest follow-up. The data included the patient’s age, gender, date of diagnosis, pathological diagnosis, initial tumor location, MRI-confirmed tumor size, and nature of the specimen types (primary or recurrent tumor). Other characteristics were *MGMT* promoter methylation status, cycles of temozolomide treatment, survival time in days after treatment, and patient status at the last follow-up (dead or alive). Missing data and patients who were lost to follow-up were not counted in the study. The study cutoff date was November 30, 2020, and patients who survived through this date were defined as the last follow-up date.

### PCR and PCR-Based Microsatellite Loss of Heterozygosity Analysis

Genomic DNA was extracted from frozen tumor tissue using PureLink Genomic DNA mini kit (Life Technologies), according to the manufacturer’s protocol. After DNA extraction, 50 ng of genomic DNA was amplified using AmpliTaq gold 360 master mix (Applied Biosystems) by PCR. Reaction mix measuring 25 μl was subjected to an initial denaturation cycle of 95°C for 7 min, followed by 35 cycles of denaturation at 95°C for 30 s, annealing at 58°C–62°C for 30 s, and extension at 72°C for 30 s, with a final extension at 72°C for 7 min.

**Figure 1:**
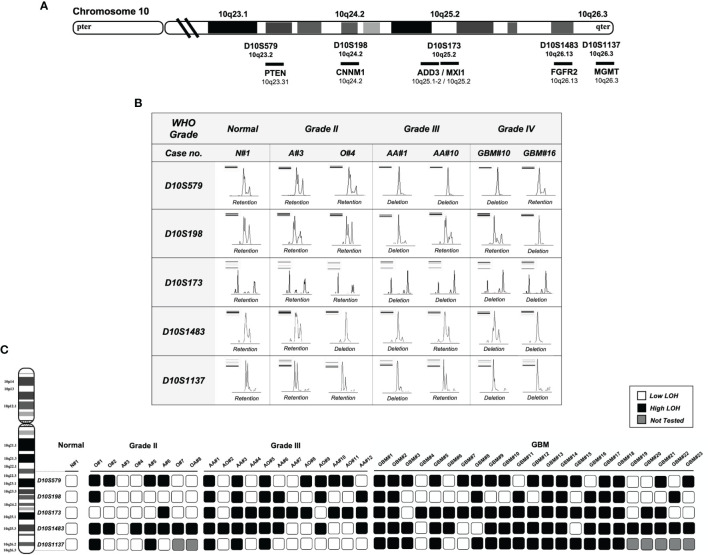
Deletion mapping of chromosome 10q using PCR-based loss of heterozygosity (LOH) analysis. **(A)** The five polymorphic microsatellite markers from 10q23 to 10q26 (D10S579, D10S198, D10S173, D10S1483, and D10S1137) and the corresponding chromosomal loci flanking or intragenic of the gene locus (chromosome map based on UCSC GRCh37/hg19 assembly). **(B)** Assessment of allelic loss in fragment analysis on an automated capillary electrophoresis system (from seven representative cases). Allelic status was calculated based on the peak intensity ratios in gliomas relative to normal brain tissue. LOH ratio of >0.5 or < 1.5 is defined as a low level of LOH, and a ratio of ≤0.5 or ≥ 1.5 is defined as a high level of LOH. DNA bands from gel electrophoresis are indicated on the top left corner of each panel. **(C)** Deletion map of 43 glioma specimens. Case numbers are indicated at the top of each column. Open and solid boxes represent low and high levels of LOH, respectively. Boxes in gray were not tested. N, normal; A, astrocytoma; O, oligodendroglioma; AA, anaplastic astrocytoma; AO, anaplastic oligodendroglioma; GBM, glioblastoma.

LOH on chromosome 10q was studied by PCR-based microsatellite analysis of the frequently deleted regions on chromosome 10q. Five microsatellite markers spanning across 10q23-qter were selected according to the genetic loci on the UCSC Table Browser on Human (GRCh37/hg19) assembly. Primer sequences were purchased from Life Technologies. Microsatellite marker D10S579 flanked *PTEN* gene (10q23.2); D10S198 is an intragenic marker within *CNNM1* gene (10q24.2); D10S173 is intragenic within *MXI1* and is 50 kb adjacent to *ADD3* gene (10q25.2); D10S1483 is intragenic of *FGFR2* gene (10q26.13); and D10S1137 flanked *MGMT* gene (10q26.3). The cytogenetic localization and the approximate genetic distances are shown in **Figure 1**. Tumor DNA samples were analyzed by capillary gel electrophoresis. DNA fragments from the PCR reaction were separated by a Fragment Analyzer automated CE system (Agilent). Allelic loss for each microsatellite locus was determined by the evaluation of peak intensity using PROSize 3.0 software (Agilent).

### Loss of Heterozygosity Evaluation

Allelic loss was evaluated according to the peak–height ratio as previously described (23). A high level of LOH was assumed when the signal intensity of the allele in tumor tissue was less than half of that in the reference DNA (i.e., DNA from the normal brain). LOH ratio ≤0.5 or LOH ratio ≥1.5 is defined as a high level of LOH; LOH ratio >0.5 or <1.5 is defined as low level or no LOH. LOH ratio of <0.5 indicates significant loss of allele 2 in the glioma tissue, and a ratio of >1.5 indicates the significant loss of allele 1 in the glioma tissue. LOH10q was defined when at least three informative loci were deleted.


$$LOH\;ratio=\frac{Peak\;height(Normal\;allele\;1)}{Peak\;height(Normal\;allele\;2)}÷\frac{Peak\;height\;(Glioma\;allele\;1)}{Peak\;height\;(Glioma\;allele\;2)}$$

### Bioinformatics Analysis

Gene expression data, DNA copy number information, and survival data were downloaded from cbioportal for cancer genomics (https://www.cbioportal.org/). The Cancer Genome Atlas (TCGA) project used for analyses consisted of clinical and gene expression data from 206 GBM patients (24).

### Western Blotting

Tissue protein lysates were prepared from clinical specimens snap-frozen in liquid nitrogen and stored at −80°C until use. Protein lysates were extracted using radioimmunoprecipitation assay (RIPA) lysis buffer with protease inhibitor cocktail. Detailed procedures of immunoblotting were performed as previously described (25). Anti-*ADD3* antibody was purchased from Sigma-Aldrich (#HPA035696); anti-*GAPDH* antibody (#2118) was purchased from Cell Signaling Technologies. Protein band intensities were quantified by ImageJ software. Band intensities were measured as the total volume under the peak of interest, with background intensity subtracted under the peak of interest, and normalized to that of the reference protein (GAPDH).

### Immunohistochemical Staining

Immunohistochemical (IHC) staining of formalin-fixed paraffin-embedded (FFPE) sections was performed on consecutive 5-μm-thick sections. Tissue sections were subjected to deparaffinization by xylene and rehydration in serial dilutions of ethanol, followed by heat-induced antigen retrieval in 10 mM of sodium citrate (pH 6.0). Endogenous peroxidase was quenched by treatment with 3% hydrogen peroxide for 30 min, and non-specific protein binding was blocked with 10% normal goat serum (Dako) for 1 h. Sections were incubated with primary antibodies at appropriate dilutions at 4°C overnight in a moist chamber. After incubation, sections were washed with Tris-buffered saline three times, followed by incubation with horseradish peroxidase (HRP)-conjugated secondary antibodies (Dako) for 30 min. DAKO EnVision System (Dako) was used to detect signals from DAB chromogen substrate. Finally, sections were counterstained with hematoxylin (Vector Laboratories) and mounted in DPX mounting solution (BDH Laboratory). All IHC sections were quantified and evaluated as the mean value from five random ×200 microscopic fields. All calculations on tissue sections were processed and analyzed by ImageJ software.

### Statistical Analysis

The goals of the statistical analysis were to uncover the clinical association of 10qLOH by ascertaining molecular genetics and pathologic and clinical parameters. The association between variables was tested with Pearson’s chi-squared (χ^2^) test. The Kaplan–Meier method was used to estimate overall survival. The time of an event was calculated as the date of the initial pathological diagnosis until the time to death or the time of the last contact if the patient was alive or the last day of the study period. Differences in survival distributions were evaluated using a log-rank test. All the results were considered statistically significant when the two-sided *p* was <0.05.

## Results

### Patient Demographics

This is a retrospective analysis of 43 glioma cases evaluating the association between allelic loss at chromosome 10q and the clinicopathological features. **Table 1** summarizes the patient demographics. In this glioma cohort, 21 were female and 22 were male adults (mean age 53.4 ± 17.3). There were 8 grade 2 glioma (18.6%), 12 grade 3 glioma (27.9%), and 23 grade 4 GBM (53.5%). According to the anatomical location, the frontal lobe was the most common site involved (22 cases, 51%). Twelve (28.6%) patients did not receive temozolomide (TMZ) as concurrent or adjuvant therapy, 7 (16.7%) received less than 6 cycles, and 23 (54.7%) received more than 6 cycles as adjuvant therapy.

**Table 1:** Patient demographics and the association between allelic loss at five chromosomal loci on 10q and the clinicopathological features.

Characteristics		Glioma Total n (%)	D10S579		p	D10S198		p	D10S173		p	D10S1483		p	D10S1137		p
			Low LOH	High LOH		Low LOH	High LOH		Low LOH	High LOH		Low LOH	High LOH		Low LOH	High LOH	
	**Total**	43 (100%)	16 (37.2%)	27 (62.8%)		26 (60.5%)	17 (39.5%)		12 (28.6%)	30 (71.4%)		9 (20.9%)	34 (79.1%)		18 (47.4%)	20 (52.6%)	
Gender					**0.454**			**0.092**			**0.495**			**0.767**			**0.758**
	Female	21 (48.9%)	9 (56.2%)	12 (44.4%)		10 (38.5%)	11 (64.7%)		5 (41.7%)	16 (53.3%)		4 (44.4%)	17 (50.0%)		9 (50.0%)	9 (45.0%)	
	Male	22 (51.1%)	7 (43.8%)	15 (55.6%)		16 (61.5%)	6 (35.3%)		7 (58.3%)	14 (46.7%)		5 (55.6%)	17 (50.0%)		9 (50.0%)	11 (55.0%)	
Age (years)					**0.911**			**0.146**			**0.091**			**0.296**			**0.564**
	≤50	13 (30.2%)	5 (31.2%)	8 (29.6%)		10 (38.5%)	3 (17.6%)		6 (50.0%)	7 (23.3%)		4 (44.4%)	9 (26.4%)		7 (38.9%)	6 (30.0%)	
	>50	30 (69.8 %)	11 (68.8%)	19 (70.4%)		16 (61.5%)	14 (82.4%)		6 (50.0%)	23 (76.7%)		5 (55.6%)	25 (73.6%)		11 (61.1%)	14 (70.0%)	
Tumor grade					**0.573**			**0.207**			***0.000			**0.442**			***0.002
	Grade II	8 (18.6%)	4 (25.0%)	4 (14.8%)		7 (26.9%)	1 (5.8%)		7 (58.3%)	1 (3.3%)		1 (11.2%)	7 (20.6%)		6 (33.3%)	2 (10.0%)	
	Grade III	12 (27.9%)	5 (31.2%)	7 (25.9%)		6 (23.1%)	6 (35.3%)		2 (16.7%)	10 (33.3%)		4 (44.4%)	8 (23.5%)		9 (50.0%)	3 (15.0%)	
	Grade IV (GBM)	23 (53.5%)	7 (43.8%)	16 (59.3%)		13 (50.0%)	10 (58.9%)		3 (25.0%)	19 (63.4%)		4 (44.4%)	19 (55.9%)		3 (16.7%)	15 (75.0%)	
Tumor location					**0.942**			**0.631**			**0.226**			**0.661**			**0.860**
	Frontal	22 (51.1%	9 (56.2%)	13 (48.1%)		13 (50.0%	9 (52.9%)		8 (66.7%)	13 (43.3%)		3 (33.4%)	19 (55.9%)		11 (61.1%)	10 (50.0%)	
	Temmporal	8 (18.6%)	3 (18.8%)	5 (18.5%)		6 (23.1%)	2 (11.8%)		3 (25.0%)	5 (16.7%)		2 (22.2%)	6 (17.6%)		3 (16.7%)	4 (20.0%)	
	Parietal	7 (16.3%)	2 (12.5%)	5 (18.5%)		3 (11.5%)	4 (23.5%)		1 (8.3%)	6 (20.0%)		2 (22.2%)	5 (14.7%)		2 (11.1%)	4 (20.0%)	
	Multiple	6 (14.0%)	2 (12.5%)	4 (14.9%)		4 (15.4%)	2 (11.8%)		0	6 (20.0%)		2 (22.2%)	4 (11.8%)		2 (11.1%)	2 (10.0%)	
Tumor size (cm)					**0.544**			**0.927**			**0.501**			**0.386**			**0.622**
	≤5	25 (65.8%)	9 (60.0%)	16 (69.6%)		15 (65.2%)	10 (66.7%)		5 (55.6%)	19 (67.9%)		7 (77.8%)	18 (64.3%)		10 (62.5%)	12 (70.6%)	
	>5	13 (34.2%)	6 (40.0%)	7 (30.4%)		8 (34.8%)	5 (33.3%)		4 (44.4%)	9 (32.1%)		2 (22.2%)	10 (35.7%)		6 (37.5%)	5 (29.4%)	
MIB-1 (%)					**0.173**			**0.148**			**0.002			**0.315**			*0.022
	<10	13 (48.1%)	6 (66.7%)	7 (38.9%)		10 (58.9%)	3 (30.0%)		10 (83.3%)	3 (21.4%)		1 (25.0%)	12 (52.2%)		9 (75.0%)	3 (27.3%)	
	≥10	14 (51.9%)	3 (33.3%)	11 (61.1%)		7 (41.1%)	7 (70.0%)		2 (16.7%)	11 (78.6%)		3 (75.0%)	11 (47.8%)		3 (25.0%)	8 (72.7%)	
MGMT promoter					**0.729**			**0.310**			**0.927**			*0.016			**0.183**
	Unmethylated	18 (50.0%)	7 (53.8%)	11 (47.8%)		9 (42.9%)	9 (60.0%)		4 (50.0%)	13 (48.1%)		1 (12.5%)	17 (60.7%)		4 (33.3%)	11 (57.9%)	
	Methylated	18 (50.0%)	6 (46.2%)	12 (52.2%)		12 (57.1%)	6 (40.0%)		4 (50.0%)	14 (51.9%)		7 (87.5%)	11 (39.3%)		8 (66.7%)	8 (42.1%)	
TMZ treatment					**0.760**			**0.290**			**0.760**			**0.518**			**0.409**
	Yes ≤ 6	7 (16.7%)	2 (13.3%)	5 (19.2%)		3 (12.0%)	4 (23.5%)		2 (16.8%)	5 (17.3%)		1 (12.5%)	6 (17.6%)		4 (22.2%)	3 (15.8%)	
	Yes > 6	23 (54.7%)	8 (53.3%)	15 (57.7%)		15 (60.0%)	8 (47.1%)		5 (41.6%)	17 (58.6%)		6 (75.0%)	17 (50.0%)		7 (38.9%)	11 (57.9%)	
	No	12 (28.6%)	5 (33.4%)	6 (23.1%)		7 (28.0%)	5 (29.4%)		5 (41.6%)	7 (24.1%)		1 (12.5%)	11 (32.4%)		7 (38.9%)	5 (26.3%)	

LOH, loss of heterozygosity; TMZ, temozolomide.

*p < 0.05, **p < 0.01, ***p < 0.001.

### Allelic Loss at D10S173 and D10S1137 Is Associated With Tumor Grading and Proliferation

The associations of allelic loss at each of the 10q loci examined with clinicopathological features were analyzed by Pearson’s χ^2^ test among the cohort, and the results are summarized in **Table 1**. LOH at D10S173 and D10S1137 loci demonstrated a significant association with tumor grade (^***^ *p* < 0.001 and ^**^ *p* < 0.01, respectively), as well as the *MIB-1* proliferative index (^**^ *p* < 0.01 and ^*^ *p* < 0.05, respectively), with >10% *MIB-1* being predominant in the high LOH group (78.6%). Only D10S1483 was associated with *MGMT* promoter methylation (^*^ *p* < 0.05). We did not observe any correlation with gender, age, tumor location, or TMZ treatment.

### Deletion Mapping Revealed Diagnostic Implication of D10S173 in Gliomas

Five microsatellite markers spanning 10q23 to 10q26 were selected for LOH analyses in 43 cases of gliomas. **Figure 1A** illustrates the corresponding chromosomal loci and the markers flanking or intragenic of the gene loci. LOH with three or more loci in all examined chromosomes was considered to amount to the entire loss of the long arm (55.8% in all cases, 37.5% in grade 2, 50% in grade 3, and 69.5% in GBM); 18/43 (41.8%) showed partial or interstitial allelic losses, and only one grade 2 glioma A#3 was intact. Of the gliomas, 97% harbor allelic loss in at least one locus. The peak intensity from normal brain tissue (N#1) was used as a reference for LOH analysis as shown in **Figure 1B**. It shows the representative peak from capillary electrophoresis, where all the five alleles were lost in one of the grade 3 gliomas (AA#1). The frequency of loss in gliomas of different malignancy grades is represented as a deletion map according to their genetic loci (**Figure 1C**). Among the five microsatellite markers, D10S173 at *ADD3/MXI1* locus demonstrated frequent deletion in high-grade gliomas (83.3% in grade 3 and 82.6% in grade 2) and was less common in low-grade cases (12.5%) (**Table 2**). The findings suggest the diagnostic potential of allelic deletion, specifically at D10S173, for high-grade gliomas.

**Table 2:** Primer sequences and frequencies of LOH at five microsatellite markers.

Microsatellite marker	Location	Size range (bp)	Forward primer 5′–3′	Reverse primer 5′–3′	LOH		
					Grade II	Grade III	Grade IV
D10S579	10q23.2	260–276	CCGATCAATGAGGAGTGCC	ATACACCCAGCCAATGCTGC	50.0%	58.3%	69.6%
D10S198	10q24.2	184–203	TGAGGGACTCATCTTCTGTT	GTCTGTGATCCCCATGTTAG	12.5%	50.0%	43.5%
D10S173	10q25.2	155	GCTGATTTTTCCTGCTGGTC	TGTTTCTGAAGCATTTTCCTTG	12.5%	83.3%	82.6%
D10S1483	10q26.13	130–158	CAATGCTATCCCGGCTATG	TCAAGACTGCAAGCGTGT	87.5%	66.7%	82.6%
D10S1137	10q26.3	600	GGAGACAGAGCAAGACCTG	GATGACTCTCCAGCAGCTTC	33.3%	25.0%	83.3%

LOH, loss of heterozygosity.

### Copy Number Deletion of ADD3 and MXI1 at D10S173 Locus Is Associated With Poor Survival

**Figure 2:**
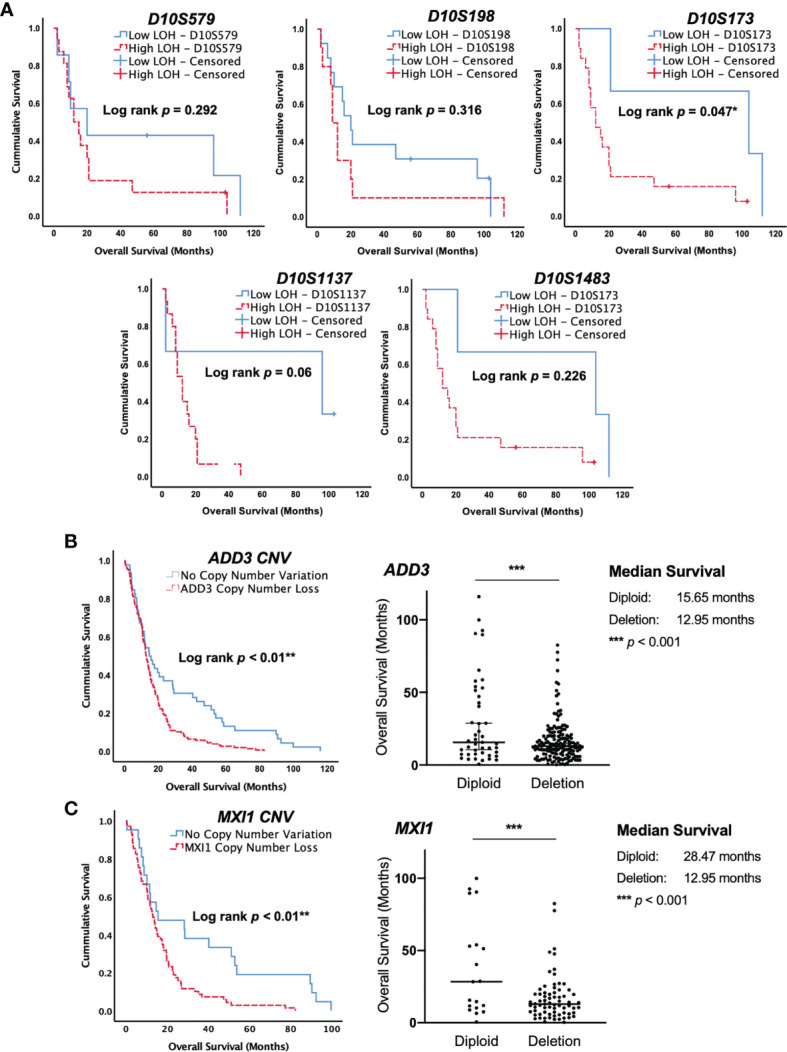
Allelic loss at the *ADD3* locus is associated with poor survival in glioblastoma (GBM). **(A)** Kaplan–Meier survival curve in our cohort of GBM. Allelic loss at D10S579, D10S198, D10S1483, and D10S1137 showed no significant difference in patient survival. A high level of deletion at D10S173 was statistically correlated with shorter survival compared with patients that had low-level deletion (^*^ *p* < 0.05). **(B, C)** The Cancer Genome Atlas (TCGA) database with 203 GBM specimens was used for validation, and data were consistent with those in our cohort, suggesting that a copy number loss of *ADD3* and *MXI1* (D10S173) is associated with a worse clinical outcome (^**^ *p* < 0.01). *ADD3* copy number variation in 202 GBM and the overall patient survival. A total of 157 samples harbor copy number loss, and 45 were diploid. Median with 95% CI (^***^ *p* < 0.0001). *MXI1* copy number variation in 90 GBM and the overall patient survival. A total of 71 samples harbor copy number loss, and 19 were diploid. Median with 95% CI (^***^ *p* < 0.0001).

The clinical outcomes of GBM patients in our cohort were examined using the Kaplan–Meier survival analysis. Log-rank test was calculated for the statistical significance between low LOH and high LOH groups. Despite the limitation of the small sample size in our study, statistical significance was achieved in GBM with D10S173 deletion. Patients with high LOH at D10S173 had poorer survival than patients with low LOH (^*^ *p* = 0.047) (**Figure 2A**). Our finding was validated using a larger GBM cohort in the TCGA database, which showed copy number variations of both *ADD3* and *MXI1* in GBM. Survival analysis of GBM with *ADD3* copy number loss, 46 diploid vs. 157 deletion (**Figure 2B**) and *MXI1* copy number loss, and 21 diploid vs. 69 deletion (**Figure 2C**) showed a significant correlation with an unfavorable outcome (log rank ^**^ *p* < 0.01), with a median survival of 12.95 months in *ADD3*-deleted tumors compared with 15.65 months in non-deleted (diploid) tumors (^***^ *p* < 0.001) and median survival of 12.95 months in *MXI1* deleted tumors compared with 28.47 months in non-deleted (diploid) tumors (^***^ *p* < 0.001).

### ADD3 Loss Is Implicated in Disease Progression

**Figure 3:**
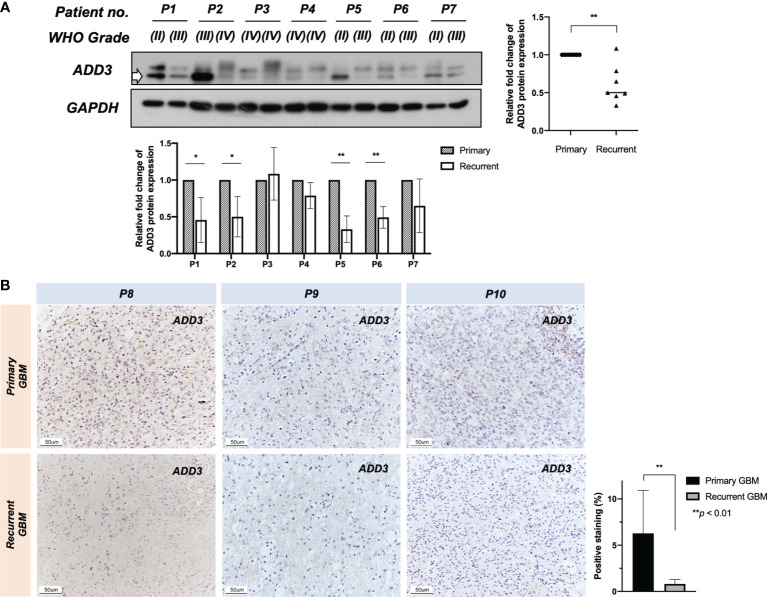
Further loss of *ADD3* expression during tumor progression. *ADD3* expression was determined in 10 patient-paired primary–recurrent gliomas (P1–P10) with different malignancy grades (WHO grade). **(A)** Western blotting analysis on seven (P1–P7) recurrent pairs and quantified signal intensity of *ADD3* protein bands (bottom band) normalized by *GAPDH* expression (bottom chart). Six out of seven recurrent gliomas had a further loss of *ADD3* expression as compared with its primary tumor (graph on the right). **(B)** Representative immunohistochemical (IHC) staining of *ADD3* in three primary–recurrent glioblastomas (GBMs) (P8–P10) formalin-fixed paraffin-embedded (FFPE) specimens, quantified with staining intensity in three individual tissue specimens for each of the tumors. Original magnification ×200 (scale bar 50 ± μm). Data represent the mean ± SD from triplicates (^*^ *p* < 0.05, ^**^ *p* < 0.0).

While the tumor-suppressive role of *MXI1* in glioma has been reported, less is known about the role of *ADD3* in glioma. In this study, we showed that LOH at D10S173 (*ADD3/MXI1*) was frequently seen in high-grade gliomas compared with low-grade gliomas and with a high proliferative index, and the results were suggestive of the role of these genes in regulating tumor progression. *ADD3* protein expression was determined by Western blotting and IHC in another cohort with 10 patient-paired primary–recurrent gliomas (P1–10). In line with our hypothesis, further losses of *ADD3* protein expression were observed in 6 out of 7 gliomas progressing to a higher malignancy grade (P1–P7, **Figure 3A**). *ADD3* was also further downregulated in recurrent GBM compared with its primary lesion (P8–P10) (**Figure 3B**).

## Discussion

Loss of genetic material at certain chromosomal regions has been considered a major event in tumor development and progression. The assessment of allelic loss is one of the most useful approaches hinting at the loci of potential tumor suppressor genes. Chromosome 10q has been suggested to encompass multiple tumor suppressors apart from *PTEN* (10q23), on account of the frequent losses observed also at loci in the distal region of 10q (10q25-qter) (5, 9, 10, 16). In an attempt to assess the possible implications of deletion at different chromosome regions, we examined the frequency of 10q LOH in a glioma cohort and evaluated the association with patients’ clinicopathological characteristics.

The results from deletion mapping were consistent with those of other studies, where most GBM cases demonstrated a high incidence of allelic loss and appeared to have lost the entire arm of chromosome 10q (69.5%). Of the five allelic loci examined, only LOH of D10S173 at the *ADD3/MXI1* locus was a predictor of shorter survival and had a significant association with tumor grade and proliferative index. *MXI1* is a transcription repressor of *MYC* (26). Despite that somatic mutations were not found in *MXI1*, which did not seem to support the two-hit hypothesis for gene inactivation as a tumor suppressor, its growth-suppressive function has been revealed in different cancers (27, 28). Compared with *MXI1*, less is known about the role of *ADD3* in cancer. We reported previously on the putative tumor-suppressive function of *ADD3* in GBM (22). It is by chance mapped to chromosome 10q25.2, where LOH frequently occurs. *ADD3* was found to be significantly downregulated in GBM, and such loss was associated with enhanced tumor growth (22). In this study, we were able to demonstrate the mechanism by which *ADD3* was downregulated in gliomas as being most likely due to LOH with copy number deletions. Our findings further suggested that *ADD3* is a putative tumor suppressor in GBM. However, one should note that the current study was conducted before the release of the fifth edition of WHO classification of gliomas published in 2021, which has grouped tumors according to the genetic changes including IDH mutation (29). The term “glioblastoma” has been classified as a specific entity with an IDH wild-type genotype. It is important to note that the term “glioblastoma or GBM” being used in this study is irrespective of the IDH mutation status. Future studies should further refine the prognostic significance of ADD3 in subgroups of tumors with and without IDH mutation.

ADD3 is a crucial assembly factor in the actin cytoskeleton that functions to recruit and promote the formation of spectrin-actin membrane skeleton to provide physical support in the cell (30, 31). Given that the cytoskeleton structures are essential for cell motility, downregulation of ADD3 has been shown to be associated with enhanced migratory and invasive potential in lung cancer cells (32). Alteration of ADD3 may also modulate the tumor microenvironment mediated by changes in focal adhesion as well as cell–cell contacts (32, 33). It was found that ADD3-deficient GBM cells were able to elicit pro-angiogenic signals to stimulate VEGFR expression in endothelial cells (22). All of these could be the potential mechanisms and the pathophysiological consequences that underlie allelic loss of ADD3 in malignant gliomas, particularly during disease progression.

There is accumulating evidence on the prognostic significance of *ADD3* in GBM. A recent study by Navarro *et al.* reported a strong association between *EGFR* amplification (or *EGFRvIII* expression) and *ADD3* copy number variations in GBM. Genetic clustering suggested the co-occurrence of both events in a subgroup of GBM, the cluster where patients were presented with the shortest survival (34). In accordance with this finding, GBMs with *EGFR* amplification are often accompanied by the complete loss of chromosome 10 and are considered the common phenotypic endpoint of different genetic pathways (35, 36). Another notable finding is the progressive event of *ADD3* loss during malignant progression in the patient-paired specimens. Indeed, *ADD3* deletion appears to occur at a much higher frequency, primarily in the most malignant subtype compared with those in low-grade cases, suggesting that further loss of *ADD3* is an event associated with disease progression. In line with this observation, LOH at 10q-qter was associated with increased proliferative activity, together with the acquisition of a morphological transition to an advanced malignant status (18).

## Conclusion

This study provides novel insights into understanding the pathogenesis of malignant progression in glioma. The identification of *ADD3/MXI1* locus carries diagnostic and prognostic implications and can be particularly helpful for making decisions concerning postoperative management. *ADD3* is a putative tumor suppressor that may also serve as a promising marker on 10q in predicting survival. While a limited number of allelic loci are being tested for LOH10q, pathological tests could be refined with additional markers at loci with higher prognostic significance. The importance of ADD3 gene assumes a new dimension that evaluation on ADD3 expression may be useful to identify patients who are likely to have a progressive disease. The fact that *ADD3* is dysregulated at the genetic level should be further explored in regard to the underlying mechanisms of tumor progression and genetic vulnerabilities in glioma. Future studies may target *ADD3* depleted tumors as a novel therapeutic approach utilizing synthetic lethality, which can be developed to target gene defects in tumors with *ADD3* deletion, similar to the concept of using *PARP* inhibitors exclusively for *BRCA*-mutated breast tumors. Future investigations may also focus on the genetic alterations associated with *ADD3* and the possible regulatory mechanisms on the transcriptional and post-transcriptional levels.

## Data Availability Statement

The raw data supporting the conclusions of this article will be made available by the authors, without undue reservation.

## Ethics Statement

The studies involving human participants were reviewed and approved by the Institution Review Board of the University of Hong Kong/Hospital Authority Hong Kong West Cluster. Written informed consent to participate in this study was provided by the participants’ legal guardian/next of kin.

## Author Contributions

KK carried out the experiments and wrote the paper. SS performed the statistical analysis and collected the data. GL conceived the study and contributed to the critical revision. All authors contributed to the article and approved the submitted version.

## Conflict of Interest

The authors declare that the research was conducted in the absence of any commercial or financial relationships that could be construed as a potential conflict of interest.

## Publisher’s Note

All claims expressed in this article are solely those of the authors and do not necessarily represent those of their affiliated organizations, or those of the publisher, the editors and the reviewers. Any product that may be evaluated in this article, or claim that may be made by its manufacturer, is not guaranteed or endorsed by the publisher.
